# An Enhanced Region Proposal Network for object detection using deep learning method

**DOI:** 10.1371/journal.pone.0203897

**Published:** 2018-09-20

**Authors:** Yu Peng Chen, Ying Li, Gang Wang

**Affiliations:** 1 College of Computer Science and Technology, Jilin University, Changchun, People’s Republic of China; 2 Key Laboratory of Symbolic Computation and Knowledge Engineering of Ministry of Education, Jilin University, Changchun, People’s Republic of China; Northeast Normal University, CHINA

## Abstract

Faster Region-based Convolutional Network (Faster R-CNN) is a state-of-the-art object detection method. However, the object detection effect of Faster R-CNN is not good based on the Region Proposal Network (RPN). Inspired by RPN of Faster R-CNN, we propose a novel proposal generation method called Enhanced Region Proposal Network (ERPN). Four improvements are presented in ERPN. Firstly, our proposed deconvolutional feature pyramid network (DFPN) is introduced to improve the quality of region proposals. Secondly, novel anchor boxes are designed with interspersed scales and adaptive aspect ratios. Thereafter, the capability of object localization is increased. Thirdly, a particle swarm optimization (PSO) based support vector machine (SVM), termed PSO-SVM, is developed to distinguish the positive and negative anchor boxes. Fourthly, the classification part of multi-task loss function in RPN is improved. Consequently, the effect of classification loss is strengthened. In this study, our proposed ERPN is compared with five object detection methods on both PASCAL VOC and COCO data sets. For the VGG-16 model, our ERPN obtains 78.6% mAP on VOC 2007 data set, 74.4% mAP on VOC 2012 data set and 31.7% on COCO data set. The performance of ERPN is the best among the comparison object detection methods. Furthermore, the detection speed of ERPN is 5.8 fps. Additionally, ERPN obtains good effect on small object detection.

## Introduction

Recently, the object detection [1–5] problems are one of the key tasks in the computer vision field. Region proposals are applied by most of the top-performing object detection methods to search for objects. A superior mean average precision (mAP) value is achieved by the state-of-the-art Region-based Convolutional Neural Network (R-CNN) [6]. The relationship between image classification and object detection is established by R-CNN. R-CNN consists of three steps: First, Selective Search (SS) [7] method is applied to generate around 2000 category-independent region proposals. Second, the features of each region proposal are extracted by a pre-trained convolutional model [8–12]. Third, the top-level features are classified by a set of linear Support Vector Machines (SVMs) [13], [14]. However, the computation speed of R-CNN is slow because it performs a CNN forward pass for each object proposal, without sharing computation. The performance of object detection for R-CNN is improved by Fast R-CNN [15]. The reason is that Fast R-CNN combines the region proposal classification and bounding box regression tasks into one single stage. Moreover, the region of interest (RoI) pooling strategy based on the top-level features is more efficient than the R-CNN feature extracting method. In other words, multi-task training avoids managing a pipeline of sequentially-trained tasks. Nevertheless, because SS method is applied to generate region proposals in Fast R-CNN, thereafter the detection speed of Fast R-CNN is affected. Faster R-CNN [16] solves the proposal computation bottleneck of Fast R-CNN. Two processes of Faster R-CNN are presented as follows: First, SS method is replaced by RPN which is a kind of fully convolutional network [17], [18] (FCN) and can be trained end-to-end to generate detection proposals. However, the resolution of top-level feature maps is too low for object detection and classification.

Unfortunately, four problems are not solved in the studies mentioned above. Firstly, context information is not integrated with the top-level features. Thus, the quality of generated region proposals is relatively poor. Specially, the small objects are hard to be detected. Secondly, the design for the selected scales and aspect ratios of anchor boxes is not optimal. Therefore, the ability of RPN object localization is weak. Thirdly, the classifier for solving binary classification problem is not optimal. Thereupon, the classification ability of RPN is not good. Fourthly, the classification part of multi-task loss function in RPN is not reasonable. As a result, the performance of multi-task loss function is affected.

Inspired by RPN of Faster R-CNN, we propose a novel proposal generation method called Enhanced Region Proposal Network (ERPN). Four improvements are presented in ERPN. Firstly, our proposed deconvolutional feature pyramid network (DFPN) is introduced to improve the quality of region proposals. Specially, the performance of small object detection is promoted by applying the rich top-level features. Secondly, novel anchor boxes are designed with interspersed scales and adaptive aspect ratios. Thereafter, the capability of object localization is increased. Simultaneously, the object detection speed is accelerated with only 200 top ranked proposals. Thirdly, a particle swarm optimization (PSO) based support vector machine (SVM), termed PSO-SVM, is developed to distinguish the positive and negative anchor boxes. Thereupon, the classification ability of ERPN is strengthened. Fourthly, the classification part of multi-task loss function in RPN is boosted. Consequently, the effect of classification loss is strengthened.

In this paper, excellent experiment results are achieved by our proposed ERPN on both PASCAL VOC and COCO data sets. Furthermore, the detection speed of ERPN is 5.8 fps which is faster than other methods. In other words, the performance of our ERPN based Faster R-CNN method is outstanding.

## Related work

In this section, the existing object detection methods that related to our work are reviewed. In computer vision field, traditional methods [19–21] and deep learning based methods [22–28] are applied to solve the object detection problems.

Scale-Invariant Feature Transform (SIFT) [29], Histograms of Oriented Gradient (HOG) [30] and Deformable Part Models (DPM) [31] are state-of-the-art traditional methods. Distinctive invariant features are extracted by SIFT method and then these features are applied to perform reliable matching between different views of an object or scene. The HOG is a feature descriptor counting occurrences of gradient orientation in localized portions of an image. This method is similar to the SIFT, but differs in that it is computed on a dense grid of uniformly spaced cells and uses overlapping local contrast normalization for improved accuracy. DPM can capture significant variations in appearance. Generally, image descriptors are applied by DPM to find regions with a class-specific maximum response.

Nevertheless, the traditional methods severely depend on the prior knowledge of the designer. Meanwhile, traditional methods perform poorly in terms of accuracy and speed. In recent years, deep learning based methods have achieved a huge success in the aspect of object detection. Particularly, the capability of localization and classification for deep learning based methods is enhanced by using the region proposals [32–35]. With the great success of the deep learning based methods on object detection, several works based on CNN have been designed. In R-CNN, category-independent region proposals are generated by SS method from the input image. Next, the bounding box regression and classification are executed based on the extracted feature maps to discriminate the target objects. Furthermore, Fast R-CNN has been developed to improve the computational efficiency and detection accuracy. The training process is a single-stage process that jointly learns to classify object proposals and refine their spatial locations. Moreover, RoI pooling strategy is applied to the top-level features. To further reduce the time of generating region proposals, Faster R-CNN introduces a novel RPN, which is introduced to share full-image convolutional features with the detection network, thus enabling nearly cost-free region proposals. Simultaneously, RPN is trained end-to-end to generate high-quality region proposals, which are used by Fast R-CNN for detection.

Besides, other object detection methods have also obtained good results. A bounding box regression scheme by MR-CNN [36] is designed to search objects, where bounding boxes are evaluated twice. Both inside and outside the region of interest information is exploited by ION [37] for object detection. Contextual information outside the region of interest is integrated using spatial recurrent neural networks. Inside, skip pooling is used to extract information at multiple scales and levels of abstraction. HyperNet [38] is primarily based on an elaborately designed Hyper Feature which aggregates hierarchical feature maps first and then compresses them into a uniform space.

The rest of this paper is organized as follows. Firstly, the concept of support vector machine and particle swarm optimization is introduced. Secondly, the improvements of ERPN method are presented. Thirdly, the experiment results and discussions are described. Finally, we draw some conclusions for this paper.

## Literature review

### Particle swarm optimization

The PSO algorithm is a meta-heuristic optimization technique [39] that simulates social behavior of birds flocking including the situation of the birds randomly searching for food. In general, if the birds are closer to the food, then these birds can find the food faster. In other words, the optimal method for searching food is to guide other birds to follow the birds that nearest to the food. PSO is developed based on this phenomenon and applied to settle the optimization problems. In PSO, each particle represents a solution in the searching area. In the process of problem solving, a population of particles explores the problem area. The fitness value for each particle is calculated by the fitness function. Because the population of particles can be updated based on the fitness value, therefore the particles could close to the optimal solution regions. Each particle can be guided by the velocity to fly in the searching area. As a result, most of the particles could follow the current optimum particle in the solution regions. The mathematical expression of PSO can be described as follows.

The *i*th particle in a *N*-dimensional region can be expressed as *x*_*i*_ = (*x*_*i*1_,…, *x*_*in*_,…, *x*_*iN*_). The former optimal position of the *i*th particle can be expressed as *p*_*i*_ = (*p*_*i*1_,…, *p*_*in*_,…, *p*_*iN*_), which achieves the best fitness value and is defined as *pbest*. The symbol *g* is applied to represent the index of the best *pbest* in the whole particles. Therefore this index is defined as *gbest*. The velocity of the *i*th particle is expressed as *v*_*i*_ = (*v*_*i*1_,…, *v*_*in*_,…, *v*_*iN*_). The Eq (1) shows the updating strategy of the velocity and location particle in PSO.

$$\begin{array}{l} v_{in}(k)=wv_{in}(t)+c_{1}r_{1}(p_{in}-x_{in}(t))+c_{2}r_{2}(p_{gn}-x_{in}(t))\\ x_{in}(k)=x_{in}(t)+v_{in}(k) \end{array}$$ (1)

where *t* represents the generation number; variable *k* is the (*t*+1)th generation; the *v*_*in*_(*t*) means the velocity of the *i*th particle on the *n*th dimension in *t*th generation; the weight *w* are the inertia coefficient; the value *c*_1_ and *c*_2_ are learning rates; variables *r*_1_ and *r*_2_ are random value in [0, 1]. The operation of PSO stops if the maximum iteration number or the fitness threshold is reached. The termination criterion for iterations is determined according to the maximum generation or the designated value of the fitness.

### Support vector machine

The SVM is a well-known machine learning method. Specially, SVM has a robust theoretical basis and can find global optimal solutions using a small amount of training samples. SVM has been successfully applied to works such as object classification, object detection, pattern recognition and non-linear regression. A linear model is used by SVM to realize non-linear class boundaries based on some non-linear mapping input vectors into a multi-dimensional space. A best separating hyper plane is created in the multi-dimensional space. Thereafter, the merit of SVM is to find a particular kind of linear model, the maximum hyper plane which generates the maximum separation between decision classes. The support vectors represent the training samples that are nearest to the maximum hyper plane. As a result, the SVM can find a non-linear relationship between inputs and outputs in multi-dimensional space.

A group of training samples is defined as (*x*_1_, *y*_1_), (*x*_2_, *y*_2_),…, (*x*_*k*_, *y*_*k*_), *x*_*i*_ ∈ *R*^*n*^, *y*_*i*_ ∈ *R*. In order to solve regression problem, a linear function is introduced. The linear function is described as follows:

f(x)=wx+b (2)

To minimize the squared Euclidean norm is the most important thing we care about. In general, this problem can be expressed as a convex optimization problem.

minimize12‖w‖2subjectto{y−iwxi−b≤εwxi+b−yi≤εi=1,…,n (3)

where *ε* represents the deviations from the actually targets *y*_*i*_. The optimal generalization is achieved based on the statistics learning concept. The range of variables *ξ*_*i*_ and $\xi_{i}{}^{*}$ is defined as *ξ*_*i*_ ≥ 0 and $\xi_{i}{}^{*}\geq 0$. Therefore, we can get the following equation.

minimize12‖w‖2+C∑i=1k(ξi+ξi*)C>0subjectto{y−iwxi−b≤ε+ξiwxi+b−yi≤ε+ξi*i=1,…,n (4)

The variable *C* decides the tradeoff between the flatness of *f*(*x*) and the amount up to which deviations larger than *ε* are tolerated. The dual optimal problem is acquired by using the optimization method.

$$\begin{array}{l} \text{maximize}\;W\text{(}\alpha \text{,}\alpha^{*})=-\frac{1}{2}\sum_{ij=1}^{n}(\alpha_{i}-\alpha_{i}^{*})(\alpha_{j}-\alpha_{j}^{*})(x_{i}x_{j})-\varepsilon \sum_{i=1}^{n}(\alpha_{i}+\alpha_{i}^{*})\\ \text{subject}\;\text{to}\left\{ \begin{array}{l} \sum_{i=1}^{n}(\alpha_{i}-\alpha_{i}^{*})=0\\ 0\leq \alpha_{i},\alpha_{i}^{*}\leq C \end{array}\right.\;i=1,\ldots ,n \end{array}$$ (5)

The regression function of the SVM is achieved by fixing the above optimization problems.

$$f(x)=\sum_{ij=1}^{n}(\alpha_{i}-\alpha_{i}^{*})(x_{i}x_{j})+b$$ (6)

A small number of factors $\left(\alpha_{i}-\alpha_{i}^{*}\right)$ will be assigned to positive values through Karush–Kuhn–Tucker (KKT) conditions for quadratic programming. The non-linear problems could be fixed through transforming the samples into a high dimension space. We use the kernel function, i.e. *K*(*x*_*i*_, *x*_*j*_) = *ϕ*(*x*_*i*_)*ϕ*(*x*_*j*_) to replace the kernel function. Because the expression of non-linear mapping is not known, therefore Eqs (7) and (8) can be changed as follows:

$$\begin{array}{l} \begin{array}{l} \text{maximize}\;W\text{(}\alpha \text{,}\alpha^{*})=-\frac{1}{2}\sum_{ij=1}^{n}\left(\alpha_{i}-\alpha_{i}^{*}\right)(\alpha_{j}-\alpha_{j}^{*})K(x_{i}x_{j})\\ \;+\sum_{i=1}^{n}y_{i}(\alpha_{i}-\alpha_{i}^{*})-\varepsilon \sum_{i=1}^{n}\left(\alpha_{i}+\alpha_{i}^{*}\right) \end{array}\\ \text{subject}\;\text{to}\{\begin{array}{l} \sum_{i=1}^{n}\left(\alpha_{i}-\alpha_{i}^{*}\right)=0\\ 0\leq \alpha_{i},\alpha_{i}^{*}\leq C \end{array}\;i=1,\ldots ,n. \end{array}$$ (7)

$$f(x)=\sum_{i=1}^{n}(\alpha_{i}-\alpha_{i}^{*})K(x_{i}x_{j})+b$$ (8)

Linear kernel function (Eq (9)), polynomial kernel function (Eq (10)), the RBF kernel function (Eq (11)) and sigmoid kernel function (Eq (12)) are presented as follows:

$$\text{K}\left(x_{i},x_{j}\right)=x_{i}{}^{T}x_{j}$$ (9)

$$\text{K}\left(x_{i},x_{j}\right)={\left(\gamma x_{i}{}^{T}x_{j}\text{+}r\right)}^{d},\gamma >\text{0}$$ (10)

$$\text{K}\left(x_{i},x_{j}\right)=\text{exp}\left(-\gamma ∥x_{i}-x_{j}{∥}^{2}\right),\gamma >0$$ (11)

$$\text{K}\left(x_{i},x_{j}\right)=\text{tanh}\left(\gamma x_{i}{}^{T}x_{j}+r\right)$$ (12)

where *γ*, *r* and *d* are kernel parameters. For the purpose of improving anchor boxes classification capability, the softmax classifier is replaced by the SVM classifier.

## Improvements of ERPN

### Overview

**Fig 1:**
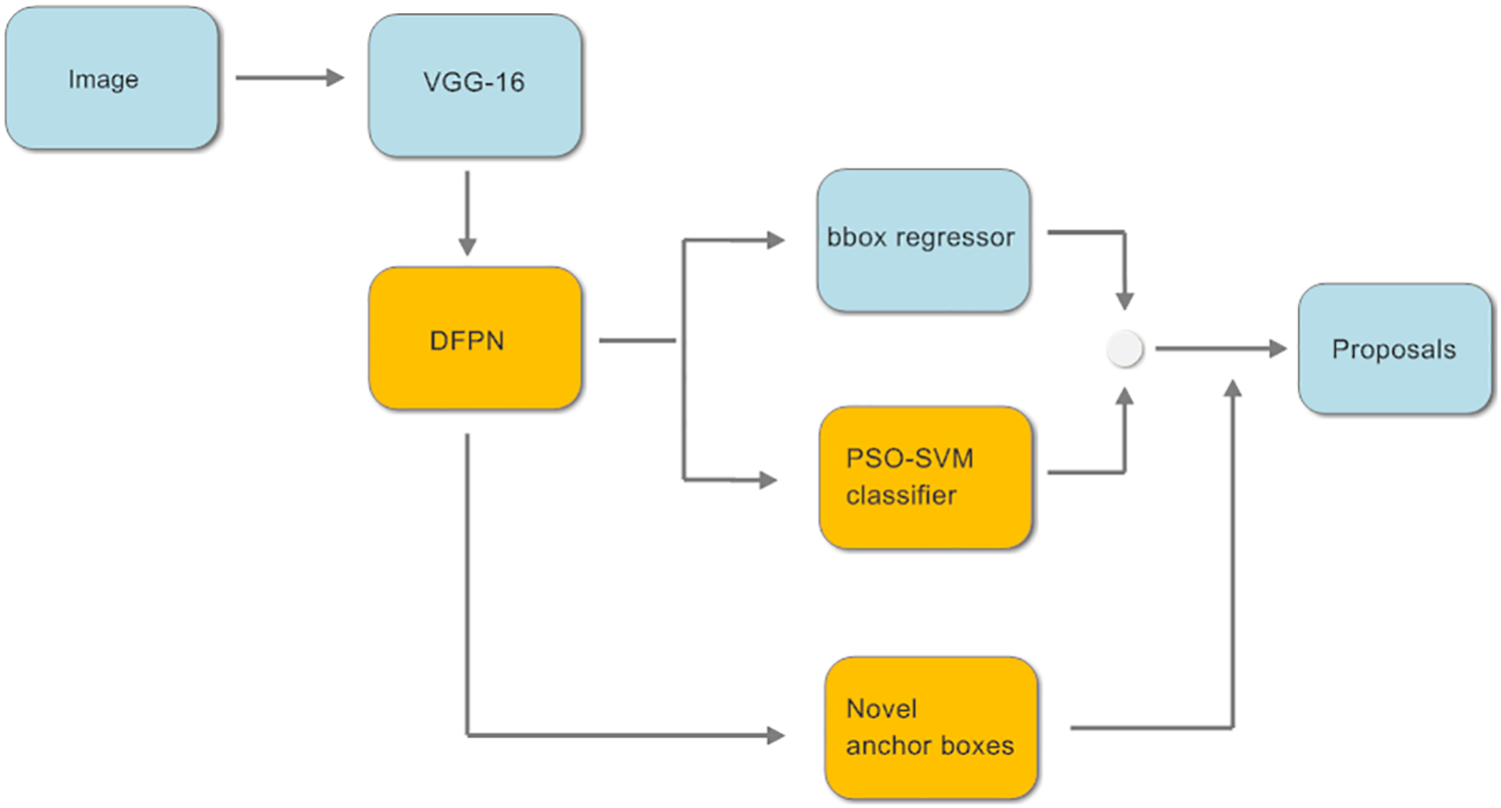
Overview of the proposed ERPN.

The architecture of our proposed ERPN is illustrated in Fig 1. Four improvements are showed with yellow boxes. The basic process is described as follows: At first, a pre-trained VGG-16 convolutional model [40] is applied to compute feature maps for the entire input image. In this work, the convolutional layers of VGG-16 are represented as conv1, conv2, conv3, conv4 and conv5 respectively. Then, multi-level features of VGG-16 model are processed by our proposed DFPN. At last, the region proposals and scores are produced based on the output feature maps of DFPN.

Four improvements are presented in this section. First, a novel DFPN is designed for combining multi-level features of VGG-16 model with context information. Second, novel anchor boxes are introduced with interspersed scales and adaptive aspect ratios. Third, PSO-SVM classifier is applied to solve the classification problem. Fourth, the classification part of multi-task loss function in RPN is improved.

### A novel deconvolutional feature pyramid network

**Fig 2:**
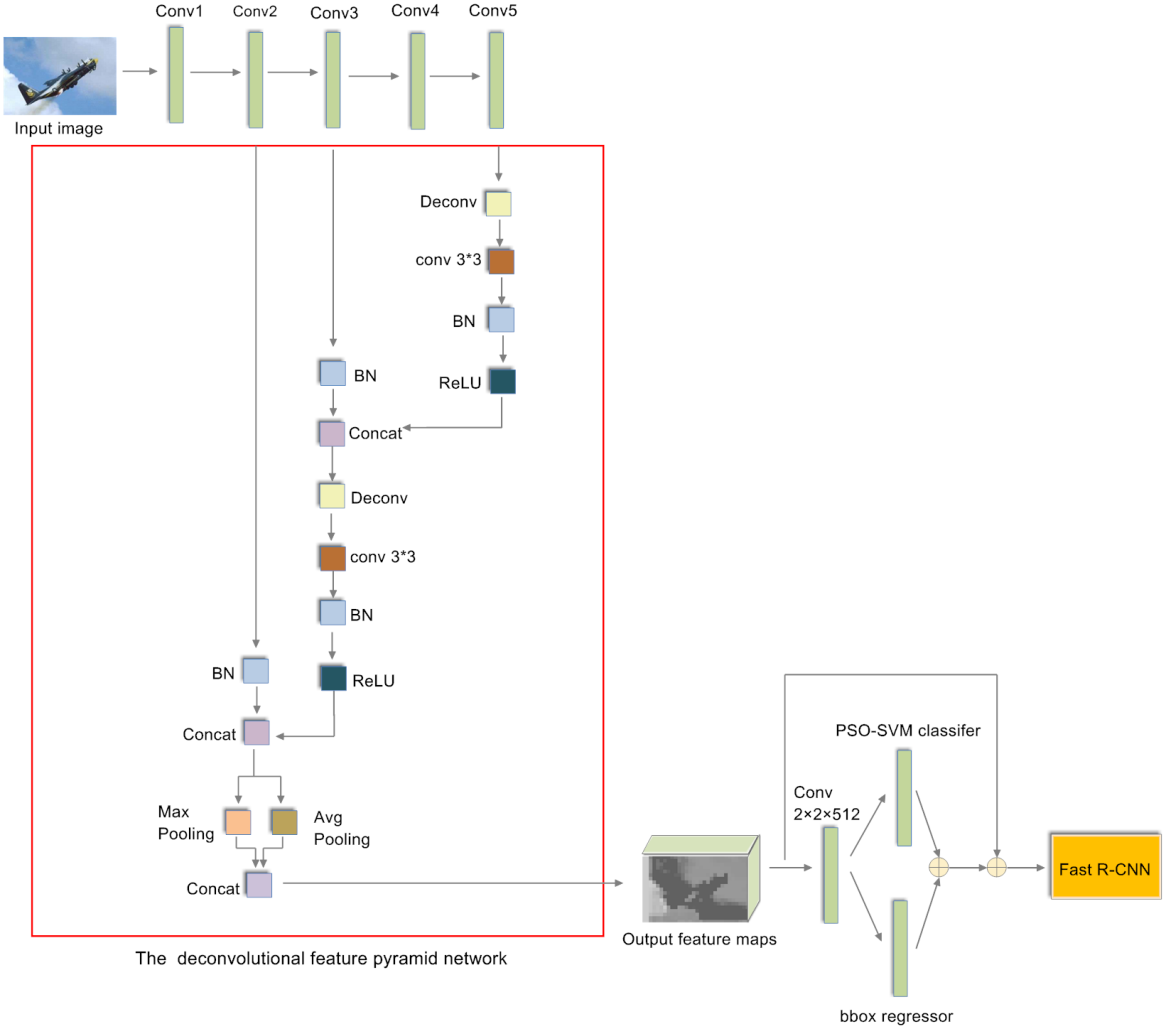
The architecture of deconvolutional feature pyramid network.

The feature maps generated by the last convolutional layer of VGG-16 are applied to predict region proposals in RPN. However, semantic information of top-level feature maps is coarse. Specially, the VGG-16 context information which contains highly semantic contents is not considered. Therefore, the quality of generated region proposals is not optimal. In addition, lower level features obtain rich information for small objects. Nevertheless, only few features of small objects are kept in the top-level feature maps. As a result, small object detection ability of Faster R-CNN is relatively poor based on the proposals generated by RPN. In order to solve the problems mentioned above, a novel DFPN is introduced. The framework of DFPN is illustrated in Fig 2.

The context information of convolutional network contains multi-level features. Specially, the resolution of lower level feature maps is large. Simultaneously, rich semantic information is kept in higher level features. Therefore, our proposed DFPN is applied to enhance the top-level features with the context information. In addition, the resolution of the feature maps for different convolutional layers is not the same. Thereafter, different methods are carried out for different layers. Our proposed methods in DFPN are described as follows.

In order to integrate lower feature maps with the higher feature maps, deconvolutional layer [41] (Deconv) is introduced into DFPN. Deconv is applied to increase the resolution of the higher convolutional feature maps. Furthermore, 3×3 convolutional layer is added on the Deconv. Therefore, more semantic features are extracted by the convolutional operation. Next, a batch normalization layer is added after each 3×3 convolutional layer. Thereafter, convolutional features are compressed into a uniform space. Meanwhile, these features are suitable for subsequent feature fusion. The above processes fit into the overall DFPN architecture as indicated in Fig 2. Additionally, because the information extracted by max pooling layer does not represent most of the features, thus the average pooling layer is an important complementary to the max pooling layer. Consequently, a synthesized pooling method including max pooling and average pooling strategies is introduced to the output concatenation features. For a resized 1000×600 input image, the resolution of output feature maps for DFPN is 125×75, which is more suitable for detection. Particularly, the ability of small object detection is strengthened by using the final output feature maps with rich information.

Feature maps of conv2, conv3 and conv5 are applied to implement feature fusion for three reasons. First, object detection is a fundamental work in vision and may need to provide information for the subsequent works. Thereafter, testing speed is an important factor. Nevertheless, the testing time is increased when the feature maps of conv1, conv2, conv3, conv4 and conv5 are applied. This result is not what we want in the real time detection system. Second, there are no pre-trained models for our proposed layers on the classification task of the ImageNet Large Scale Visual Recognition Challenge (ILSVRC) [42] dataset. Thus, our proposed layers should be trained starting from random initialization. Consequently, training time is too much when all convolutional layers are applied to DFPN. Thirdly, because the feature map of conv1 is too large, thus this feature map is not included in our proposed DFPN. As a result, the feature maps of conv2, conv3 and conv5 are applied to DFPN.

### Novel anchor boxes

For original anchor boxes [16], 3 scales with box areas of 128^2^, 256^2^ and 512^2^ pixels, and 3 aspect ratios of 1:1, 1:2 and 2:1 are used in RPN. Nevertheless, anchor boxes generation strategy for each sliding position is the same. Thus, the overlap situation of the anchor boxes for the adjacent sliding position is very serious. In other words, most of the adjacent anchor boxes with the same area and aspect ratio are invalid. As a result, the performance of object detection is affected. Simultaneously, 9 anchor boxes are generated at each sliding position. Therefore, there are around 20k anchor boxes in total for a convolutional feature map of a size *W*×*H* (typically around 2,400). Thus, our output layer has 2.8 × 10^4^ parameters (512 × (4 + 2) × 9 for VGG-16) which increase the computation time. Furthermore, the aspect ratio of each anchor box is a constant value. However, the aspect ratio of each feature map is different. Thereafter, the relationship between the aspect ratio of each anchor box and the aspect ratio of feature map is not considered. Consequently, the capability of object localization is weak.

**Fig 3:**
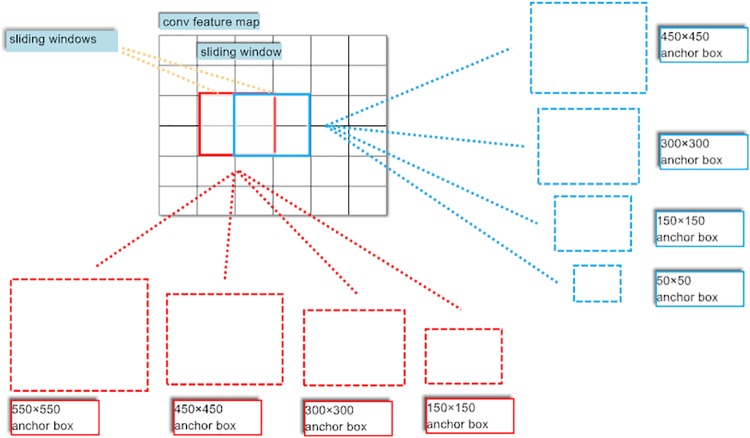
Diagram of the interspersed scales for novel anchor boxes.

In this work, novel anchor boxes are designed to solve the problems mentioned above. Two improvements are included in our proposed anchor boxes. Firstly, the anchor boxes with 4 scales are divided into two types. The areas for one type of anchor box scales are 150^2^, 300^2^, 450^2^ and 550^2^ pixels. The areas with 300^2^, 450^2^ and 550^2^ are in the range of 300^2^ to 600^2^. Meanwhile, the area with 150^2^ is in the range of 0 to 300^2^. Because the short size of input images is resized to 600, thus the anchor boxes with areas of 150^2^, 300^2^, 450^2^ and 550^2^ pixels are suitable for the larger object detection. In addition, the areas for another type of anchor box scales are 50^2^, 100^2^, 250^2^ and 400^2^ pixels. The area with 400^2^ is in the range of 300^2^ to 600^2^. At the same time, the areas with 50^2^, 100^2^, 250^2^ are in the range of 0 to 300^2^. Therefore, the anchor boxes with areas of 50^2^, 100^2^, 250^2^ and 400^2^ pixels are suitable for the smaller object detection. These two types of anchor boxes scales are interspersed for each 2×2 sliding window. Fig 3 shows the diagram of the anchor boxes with interspersed scales. Secondly, there is a relationship between the aspect ratio of the objects and the aspect ratio of input image containing objects. Particularly, the aspect ratio of each feature map is the same as the aspect ratio of input image. Therefore, we make the aspect ratio of each anchor box equal to the aspect ratio of corresponding feature map. In other words, the improved anchor boxes are adaptively matched to the input image. Consequently, the performance of object detection with our adaptively anchor boxes is promoted.

**Fig 4:**
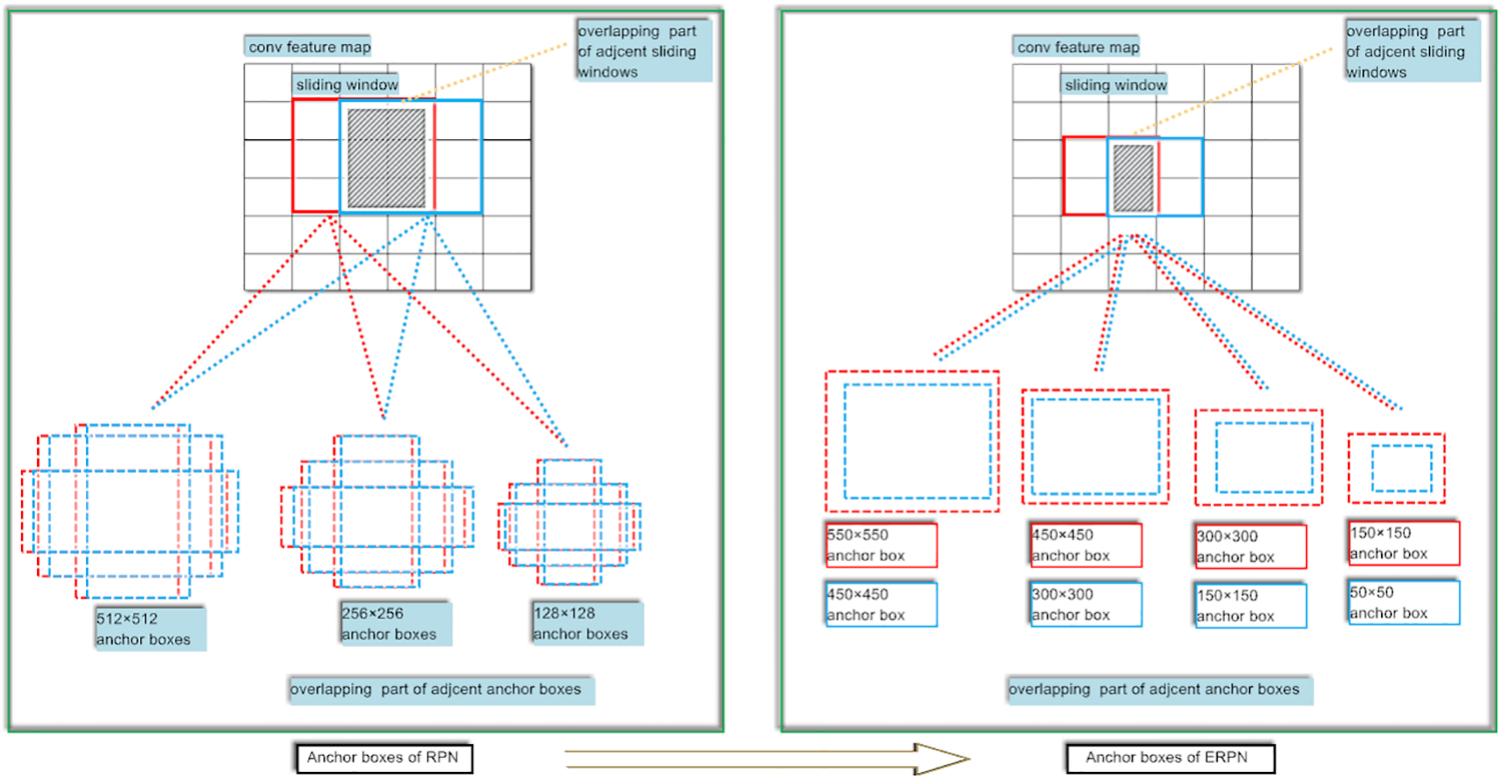
Comparison between the anchor boxes of RPN and ERPN.

The comparison between the original anchor boxes and the novel anchor boxes is showed in Fig 4. Intersection over union (IoU) is defined as (*w*∩*b*)/(*w*∪*b*) where *w* and *b* are the object proposal bounding boxes and ground truth boxes. Because the IoU for adjacent original anchor boxes with the same scale and aspect ratio is high, therefore most of the original anchor boxes are redundant. Nevertheless, because the adjacent novel anchor boxes have different areas, thus the IoU for adjacent novel anchor boxes is lower than the IoU for original anchor boxes. Specially, 4 anchor boxes are applied to each sliding position. As a result, total around 30k anchor boxes are generated for a convolutional feature map of a size *W*×*H* (around 9k). However, output layer of DFPN has 1.2 × 10^4^ parameters (512 × (4 + 2) × 4 for VGG-16) which are less than that of RPN. Thereupon, the computation speed of object detection is accelerated.

### A novel PSO-SVM classifier

SVM classifier is widely used to solve classification problems. Specially, SVM classifier is useful for multi-class classification problems. In this paper, SVM is applied to classify each region proposal in ERPN.

The optimal solution can be achieved by the kernel functions of SVM. The RBF is used frequently among these kernel functions. The parameters of RBF are relatively few. Besides, the performance of RBF and other kernel functions are nearly the same. Thereafter, RBF is a good choice for kernel function [43]. As a result, RBF is applied in our ERPN to solve classification problem.

The parameters *C* and *γ* need to be adjusted appropriately in SVM. The parameter *C* is the penalty item. The classification result can be influenced by the value of *C*. The classification accuracy fluctuates very much in the training and testing phase if *C* is too large. The effect of classification is not good if *C* is too small. The influence of parameter *γ* on classification results is much greater than parameter *C*, because the partitioning outcome is affected by parameter *γ* in the feature space. The over-fitting problem can be caused by the large value of parameter *γ*. Conversely, small value of parameter *γ* can lead to under-fitting. Because the appropriate values of parameters *C* and *γ* can promote the classification performance of SVM, then the values of parameters *C* and *γ* are set through the grid search method in the most situation. However, the global searching ability of grid search is not good. Thereafter, the classification rate of SVM is easily to fall into local optima by using grid search method. In addition, the searching interval of parameters is hard to define. The calculation resource is wasted if the searching interval is too large, while the calculation speed is affected if the searching interval is too small. In a word, the performance of SVM is seriously affected by the parameters *C* and *γ*. In order to enhance the ability of SVM, the PSO method is applied to optimize the parameters *C* and *γ* of SVM. The optimization of SVM hyperparameters is determined by two important aspects. First, each particle is constructed by two parameters *C* and *γ*. Thereupon, the flying of particles represents the changes in parameters *C* and *γ*. Secondly, the performance of each particle is evaluated by the fitness function. Therefore the local and global optima are updated based on the fitness value. The fitness of a particle is described as follows:

$$f_{i}=Ave_{\text{test}-10}$$ (13)

where *f*_*i*_ represents the fitness value of *i*th particle, and *Ave*_test-10_ means ten-fold cross validations are applied to the training samples for each particle and the average classification correct rate is used as the fitness value.

**Fig 5:**
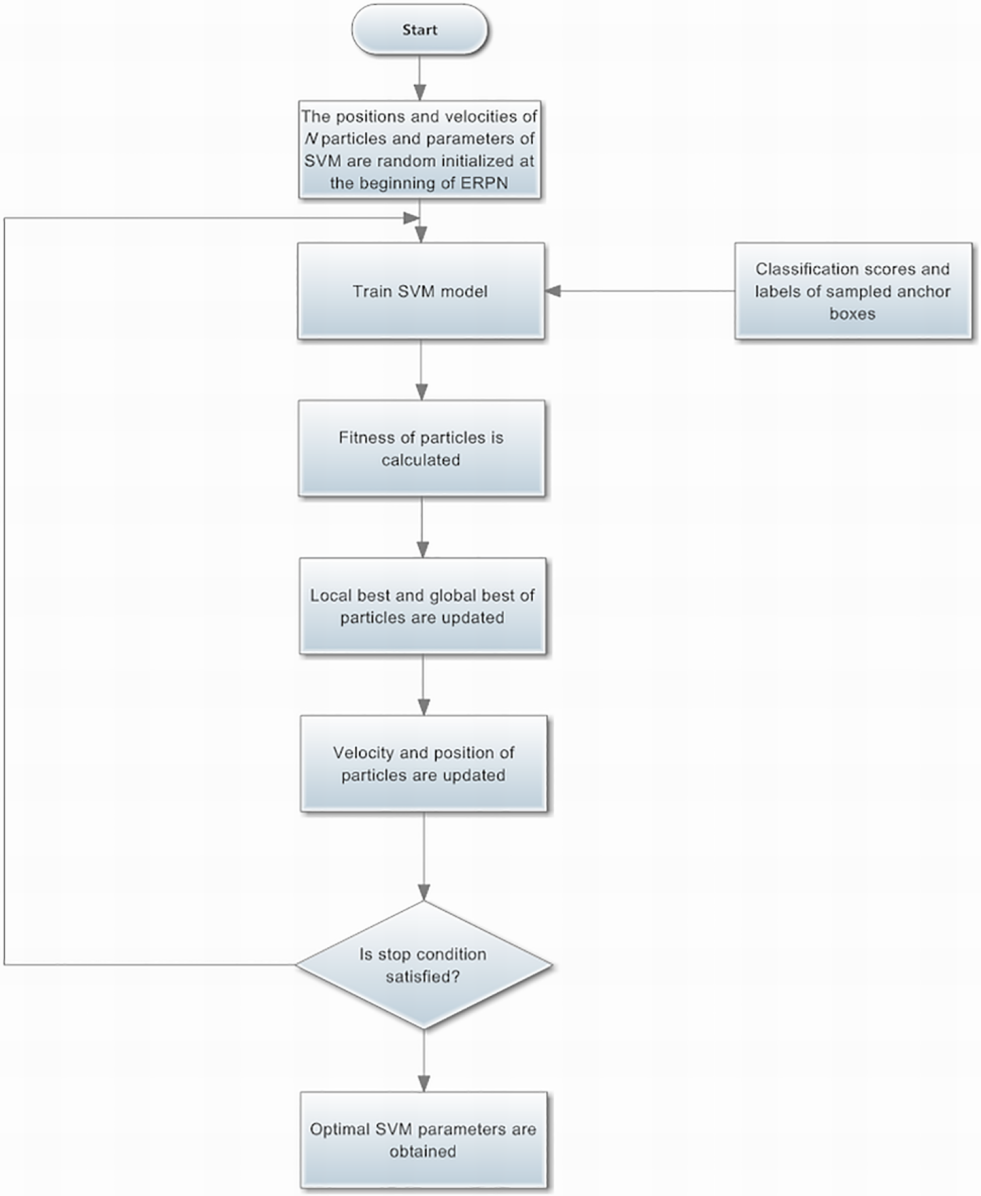
The flowchart of optimizing the SVM parameters with PSO.

The flowchart of optimizing the SVM parameters with PSO is illustrated in Fig 5. At first, the positions and velocities of *N* particles and parameters of SVM are random initialized at the beginning of ERPN. The 256 anchor boxes are randomly sampled for an image to train the SVM model. Moreover, the ratio of the positive and negative samples is 1:1. Simultaneously, the negative samples are padded the mini-batch if the number of positive samples is less than 128. Moreover, the fitness of particles is calculated. Here, each particle is composed of the parameters *C* and *γ*. Next, the local best and global best of particles are updated. Then, the velocity and position of particles are updated. Furthermore, if the iteration number of PSO is reached, then the optimization process of SVM is finished. As a result, we can obtain the optimal parameters *C* and *γ*. The searching range for parameters *C* and *γ* is [0.01, 35000] and [0.0001, 32] respectively.

**Fig 6:**
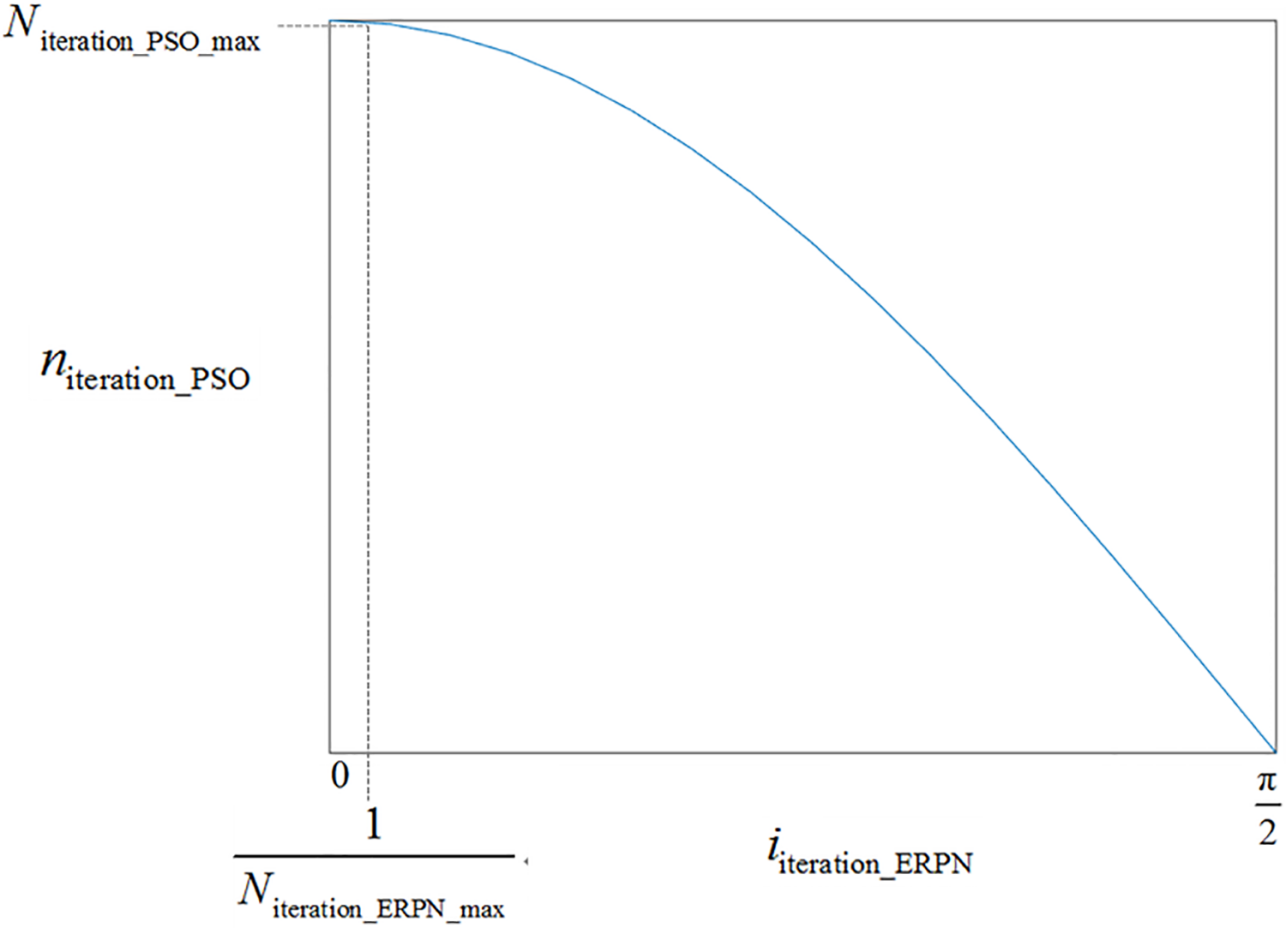
The PSO iteration number.

Because the parameters *C* and *γ* are randomly initialized at start, therefore the iteration number of PSO should be relatively large. As the parameters of SVM stabilized, the iteration number of PSO should be relatively small. In this way, the training time can be reduced as much as possible. The following equation is designed according to the above idea.

$$n_{\text{iteration}\_\text{PSO}}=N_{\text{iteration}\_\text{PSO}\_\text{max}}\times \text{cos}(\frac{i_{\text{iteration}\_\text{ERPN}}}{N_{\text{iteration}\_\text{ERPN}\_\text{max}}}\times \frac{\pi }{2})$$ (14)

where *n*_iteration_PSO_ is the PSO iteration number for each calculation of ERPN; variable *N*_iteration_PSO_max_ is the max iteration number of PSO; variable *i*_iteration_ERPN_ is the current iteration number of ERPN; variable *N*_iteration_ERPN_max_ is the max iteration number of ERPN. The curve of Eq (14) is illustrated in Fig 6.

From Fig 6, we can see that the value of *n*_iteration_PSO_ is gradually decreased when the value of *i*_iteration_ERPN_ increases. In other words, the iteration PSO iteration number for each calculation of ERPN is decreased. Moreover, the variable *i*_iteration_ERPN_ is assigned to 0 when *i*_iteration_ERPN_ equals to *N*_iteration_ERPN_max_. Thereupon, the PSO method is not executed at the last step of ERPN. Because the change for the parameters of SVM is gradually stable, therefore the Eq (14)satisfies our requirements.

### Improved classification loss function

The loss function of RPN is defined as follows:

$$L\left(\left\{p_{i}\right\},\left\{t_{i}\right\}\right)=\lambda \frac{1}{N_{reg}}\sum_{i}p_{i}^{*}L_{reg}\left(t_{i},t_{i}^{*}\right)+\frac{1}{N_{cls}}\sum_{i}L_{cls}\left(p_{i},p_{i}^{*}\right)$$ (15)

where *i* is the serial number of an anchor in a batch; the coefficient *λ* is a balancing constant; the variables *N*_*reg*_ and *N*_*cls*_ are the number of mini-batch and the anchor boxes respectively; the ground-truth label $p_{i}^{*}$ equals to 1 if the anchor box is positive, and equals to 0 if the anchor box is negative; the equation $L_{reg}\left(t_{i,}t_{i}^{*}\right)=R\left(t_{i}-t_{i}^{*}\right)$ is the regression loss where *R* is the robust loss function (smooth *L*_1_) defined in [2]. The term $p_{i}^{*}L_{reg}$ represents the regression loss is available only for positive anchors $\left(p_{i}^{*}=1\right)$ and is invalid otherwise $\left(p_{i}^{*}=0\right)$. The {*p*_*i*_} and {*t*_*i*_} are included in the outputs of the *cls* and *reg* layers respectively; the variable *t*_*i*_ means 4 parameterized coordinates of the predicted bounding box, and the ground-truth box of a positive anchor is represented by $t_{i}^{*}$; the *p*_*i*_ is the predicted probability of anchor box *i* as an object; the objects or not objects are classified by a log loss *L*_*cls*_. The definition for classification loss *L*_*cls*_ is described as follow:

$$L_{cls}(p_{i},p_{i}^{*})=-p_{i}^{*}\text{log}(p_{i})-(1-p_{i}^{*})\text{log}(1-p_{i})$$ (16)

$$\Rightarrow L_{cls}(p_{i},p_{i}^{*})=\{\begin{array}{ll} -\text{log}(p_{i}\text{)} & if\;p_{i}^{*}=1\\ -\text{log}(1-p_{i}) & if\;p_{i}^{*}=0 \end{array}$$ (17)

**Fig 7:**
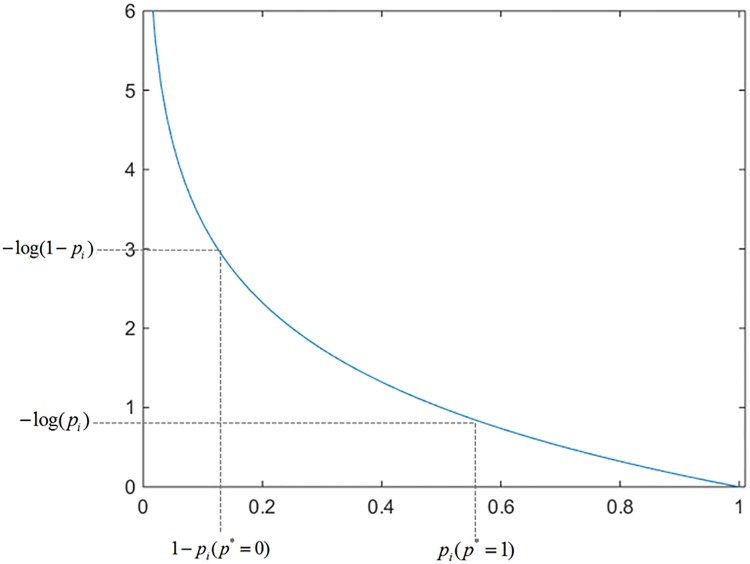
The classification loss function in RPN.

From Fig 7 we can see that if the ground-truth label $p_{i}^{*}$ equals to 1, then the *i*th anchor box is classified correctly. Therefore, the value of *p*_*i*_ is large. In other words, the value of −log(*p*_*i*_) is small. Moreover, if the ground-truth label $p_{i}^{*}$ equals to 0, then the *i*th anchor box is misclassified. In this situation the value of *p*_*i*_ is also large, thereafter the value of–log(1 − *p*_*i*_) is big. In general, number of negative samples is more than the number of positive samples. Thereafter, the effect of negative samples is bigger in the training samples. In other words, the training for negative samples is our focus.

In order to solve the problems mentioned above, a novel classification loss function is designed as follows:

$$L_{cls}(p_{i},p_{i}^{*})=-(\frac{1}{1+e^{-(-\eta p_{i})}})p_{i}^{*}\text{log}(p_{i})-(\frac{1}{1+e^{-\eta p_{i}}})(1-p_{i}^{*})\text{log}(1-p_{i})$$ (18)

$$\Rightarrow L_{cls}(p_{i},p_{i}^{*})=\{\begin{array}{ll} -(\frac{1}{1+e^{-(-\eta p_{i})}})\text{log}(p_{i}\text{)} & \text{if}\;p_{i}^{*}=1\\ -(\frac{1}{1+e^{-\eta p_{i}}})\text{log}(1-p_{i}\text{)} & \text{if}\;p_{i}^{*}=0 \end{array}$$ (19)

From Eq (19) we can see that two coefficients are applied to adjust the −log(*p*_*i*_) and–log(1 − *p*_*i*_).

**Fig 8:**
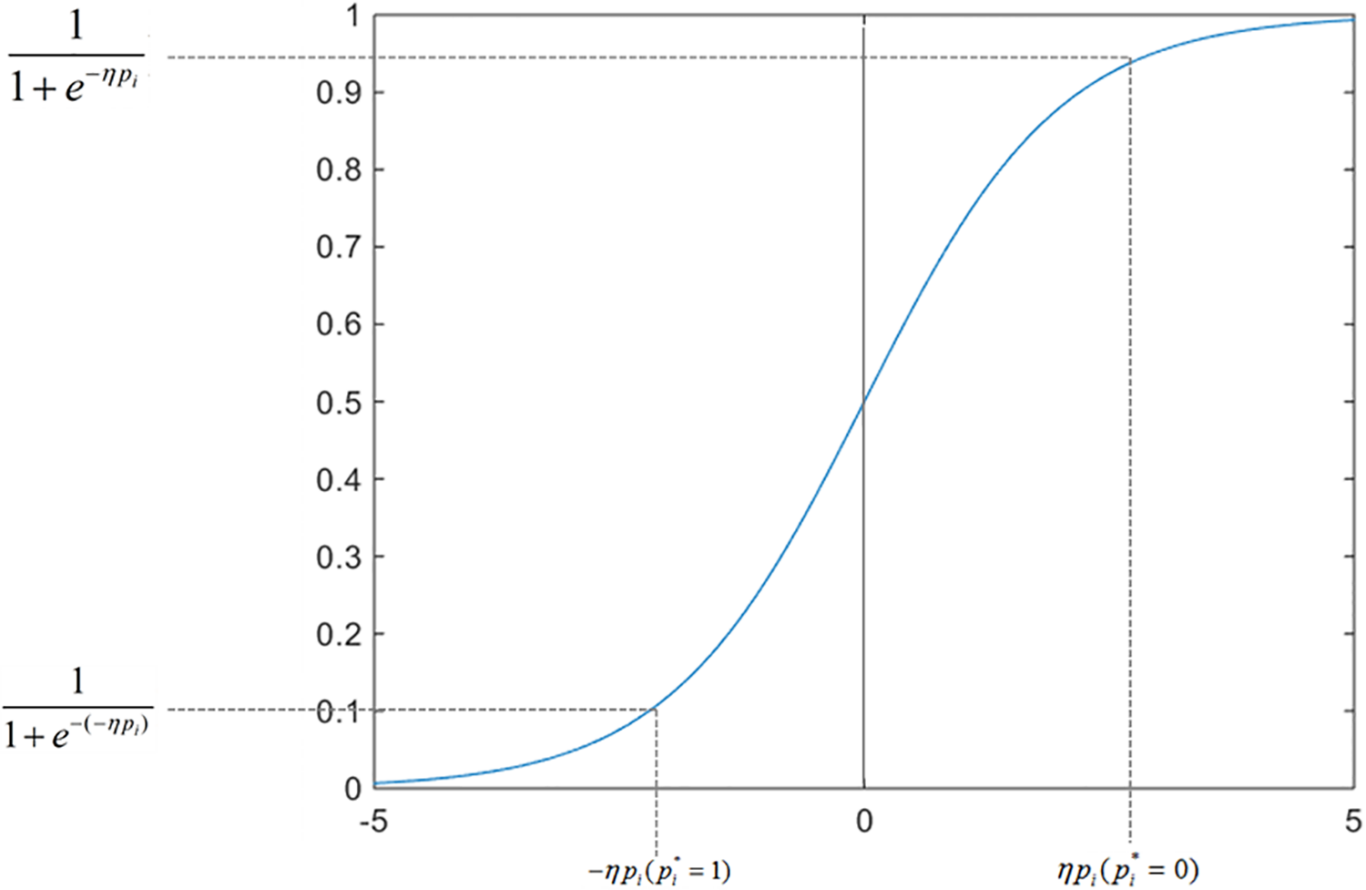
The novel coefficients of improved classification loss function in ERPN.

From Fig 8 we can find that if $p_{i}^{*}=1$ then the value of $\frac{1}{1+e^{-(-\eta p_{i})}}$ is lower than 0.5. Thus, the value of $-(\frac{1}{1+e^{-(-\eta p_{i})}})\text{log}(p_{i})$ is seriously reduced. Therefore, the effect of positive samples is diminished. On the other side, if $p_{i}^{*}=0$ then the value of $\frac{1}{1+e^{-\eta p_{i}}}$ is bigger than 0.5. Thereupon, the reduction extent of positive samples is much greater than that of negative samples. Consequently the training of negative samples is strengthened. In other words, the balance of the training samples has been improved. Additionally, variable *η* is applied to further promote the convergence effect by changing the scaling of coefficients. The value of variable *η* is assigned to 3 according to the experiment.

From Eq (1) we can see that two terms *L*_*cls*_ and *L*_*reg*_ are divided by the size of mini-batch and the number of anchor boxes. In this paper, the size of mini-batch is 256. In other words, variable *N*_*cls*_ equals to 256. Simultaneously, variable *N*_*reg*_ is about 1100 based on our novel anchor boxes. In order to make the *L*_*cls*_ and *L*_*reg*_ nearly equally weighted, variable *λ* is assigned to 4. Moreover, the value of variable *λ* is selected from 1 to 10 in our experiment. As a result, the outstanding performance of ERPN is achieved when *λ* equals to 4.

### Pseudo code of ERPN

The main task of our ERPN is to generate region proposals. In order to describe the whole process clearly, pseudo code of ERPN is introduced. The pseudo code for the training process of ERPN is showed as follows:

**[Step One]** The VGG-16 network is initialized by the pre-trained model.

**For** *i* = 1 to *maxiter* Do

**[Step Two]** A resized image is sent to VGG-16 network for forward propagation calculation.

**[Step Three]** DFPN is applied to integrate lower feature maps with the top-level feature maps.

**[Step Four]** The novel anchor boxes are generated based on the output features.

**[Step Five]** The classification and regression loss function is calculated based on the top-level features.

**[Step Six]** The back propagation for ERPN is executed.

**End**

The pseudo code for the testing process of ERPN is presented as follows:

**[Step One]** The VGG-16 network is initialized by the pre-trained model.

**For** *i* = 1 to *maxiter* Do

**[Step Two]** A resized image is sent to VGG-16 network for forward propagation calculation.

**[Step Three]** DFPN is applied to integrate lower feature maps with the top-level feature maps.

**[Step Four]** The novel anchor boxes are generated based on the output features.

**[Step Five]** The region proposals are generated by the classification and regression operations.

**End**

### Theoretical comparison between ERPN and RPN

The feature maps of last shared convolutional layer in RPN are used to generate region proposals. Nevertheless, the top-level features contain coarse information and ignore the context information in different convolutional layers. In order to integrate lower feature maps with the higher feature maps, Deconv is introduced into DFPN. Deconv is applied to increase the resolution of the higher convolutional feature maps. Therefore, the top features for proposals generation contain higher level rich semantic information and lower level high-resolution features. Specially, the resolution of top-level feature maps is enlarged. As a result, the ability of object detection is enhanced.

For original anchor boxes, 3 scales with box areas of 128^2^, 256^2^ and 512^2^ pixels and 3 aspect ratios of 1:1, 1:2 and 2:1 are used in RPN. Nevertheless, anchor boxes generation strategy for each sliding position is the same. Thus, the overlap situation for the anchor boxes of the adjacent sliding position is very serious. Simultaneously, the relationship between the aspect ratio of each anchor box and the aspect ratio of corresponding feature maps is not considered. Consequently, the capability of object localization is weak. In this work, novel interspersed anchor boxes are designed with scales of [150^2^, 300^2^, 450^2^, 550^2^] and [50^2^, 100^2^, 250^2^, 400^2^]. Therefore, the IoU for adjacent proposed anchor boxes is lower than the IoU for original anchor boxes. Simultaneously, the number of available proposals is more than that of original anchor boxes. Moreover, the aspect ratio of each anchor box is adapted to the shape of image. As a result, the performance of object detection is promoted.

Softmax classifier is used in RPN to distinguish the positive samples and negative samples. However, the SVM classifier with RBF kernel function also has strong ability to solve the binary classification problem. Specially, PSO method is applied to optimize the parameters *C* and *γ* of SVM. As a result, the classification ability of RPN is enhanced by applying the optimal SVM classifier based on the PSO method. The problem of imbalance training samples is not solved by the classification loss function of RPN. In this paper, novel coefficients of improved classification loss function are developed. In this way, the training of negative samples is strengthened. In other words, the balance of the training samples has been improved. As a result, the performance of multi-task loss function is promoted.

### Implementation details

At first, short side of the input image is resized to 600. Next, this resized entire image is sent to the region proposal generation network with the pre-trained VGG-16 model. Then, our proposed DFPN is applied to integrate lower feature maps with the top-level feature maps. Moreover, the interspersed scales of [150^2^, 300^2^, 450^2^, 550^2^] and [50^2^, 100^2^, 250^2^, 400^2^] are selected for anchor boxes. Specially, the cross-boundary anchors are ignored during training and testing. Moreover, 200 top ranked proposals are generated after NMS based on their *cls* scores. The IoU threshold for NMS is assigned to 0.75. Finally, these proposals are used for detection by Fast R-CNN.

### Time computation complexity analysis

O(*L+K+S+Q+N*) is the time computation complexity of RPN based Faster R-CNN. In this formula, *L* means the image preprocessing time; *K* shows the execution time of convolutional network; *S* represents the activity for anchor boxes generation; *Q* represents the time complexity for the multi-task loss function; *N* illustrates the detection stage with Fast R-CNN method. The time computation complexity of EPRN based Faster R-CNN can be presented as O(L+K*+S*+Q+N*). In this expression, K* represents the execution time of convolutional network with DPFN; S* is the activity for novel anchor boxes generation; N* shows the detection stage with Fast R-CNN method based on 200 input proposals. According to the previous analysis, we can see that O(*L+K+S+Q+N*)> O(L+K*+S*+Q+N*), thereupon the time computation complexity of RPN is higher than ERPN. Therefore, ERPN is more suitable for real time objection than RPN.

## Experiments

### Data sets introduction

In the experiment, the region proposals are generated by ERPN and then Fast R-CNN is applied to implement object detection with these proposals. For convenience, we use ERPN to represent the whole detection process. ERPN is trained and tested on PASCAL VOC 2007, 2012 [44] and MS COCO [45] data sets. The information of data sets is presented in Table 1. The comparison results between ERPN and the state-of-the-art object detection methods are presented. Moreover, the performance of improvements in ERPN is deeply analyzed.

**Table 1:** Data set information.

No.	Data set	No. of classes	No. of images	No. of annotated objects
1	PASCAL VOC 2007	20	9963	24640
2	PASCAL VOC 2012	20	11530	27450
3	MS COCO 2015	80	328000	2500000

Caffe [46] framework is applied to realize the ERPN. All layers of VGG-16 model is pre-trained over ILSVRC dataset. VGG-16 model contains 13 convolutional layers and 3 fully-connected layers. Recently, FCN is demonstrated impressive performance on semantic segmentation task. Inspired by these works, 3 fully-connected layers are not applied to ERPN. The mAP and recall are used to evaluate the performance of ERPN through the data sets.

### Parameter setting

In the comparative experiments, the initial parameters values of PSO, Fast R-CNN, MR-CNN, ION, ERPN, Faster R-CNN and HyperNet are presented in Tables 2–8.

**Table 2:** Parameters for PSO.

Parameter	Description	Value
*N*	Number of particles	30
*w*	Value of weight	0.7
*C*_1_ *and C*_2_	Value of Coefficients	2
*i*	Maximum number of iterations	100

**Table 3:** Parameters of Fast R-CNN.

Parameter	Description	Value
*base_lr*	Initial value of the learning rate	0.001
*lr_policy*	Learning rate policy: drop the learning rate in steps by a factor of *gamma* every *stepsize* iterations	"step"
*gamma*	Factor of dropped learning rate	0.1
*stepsize*	Drop the learning rate every *stepsize* iterations	30000
*momentum*	Weight of the previous update	0.9
*weight_decay*	Factor of the regularization	0.0005
*max_iter_fastrcnn*	Number of Fast R-CNN execution steps	40000
*IoU_ foreground_fastrcnn*	IoU value for Fast R-CNN foreground anchor boxes	[0.5,1]
*IoU_ background_fastrcnn*	IoU value for Fast R-CNN background anchor boxes	[0.1,0.5)

**Table 4:** Parameters of MR-CNN.

Parameter	Description	Value
*base_lr*	Initial value of the learning rate	0.001
*lr_policy*	Learning rate policy: drop the learning rate in steps by a factor of *gamma* every *stepsize* iterations	"step"
*gamma*	Factor of dropped learning rate	0.1
*stepsize*	Drop the learning rate every *stepsize* iterations	50000
*momentum*	Weight of the previous update	0.9
*weight_decay*	Factor of the regularization	0.0005
*max_iter*	Number of execution steps	40000
*IoU_ foreground*	IoU value for foreground anchor boxes	[0.5,1]
*IoU_ background*	IoU value for background anchor boxes	[0.1,0.5)

**Table 5:** Parameters of ION.

Parameter	Description	Value
*base_lr*	Initial value of the learning rate	0.001
*lr_policy*	Learning rate policy: drop the learning rate in steps by a factor of *gamma* every *stepsize* iterations	"step"
*gamma*	Factor of dropped learning rate	0.1
*stepsize*	Drop the learning rate every *stepsize* iterations	50000
*momentum*	Weight of the previous update	0.9
*weight_decay*	Factor of the regularization	0.0005
*max_iter_rpn*	Number of RPN execution steps	60000
*max_iter_fastrcnn*	Number of Fast R-CNN execution steps	40000
*NMS_IoU*	Threshold value of IoU for NMS method	0.443

**Table 6:** Parameters of ERPN.

Parameter	Description	Value
*base_lr*	Initial value of the learning rate	0.001
*lr_policy*	Learning rate policy: drop the learning rate in steps by a factor of *gamma* every *stepsize* iterations	"step"
*gamma*	Factor of dropped learning rate	0.1
*stepsize*	Drop the learning rate every *stepsize* iterations	50000
*momentum*	Weight of the previous update	0.9
*weight_decay*	Factor of the regularization	0.0005
*max_iter_rpn*	Number of ERPN execution steps	60000
*IoU_ foreground_ERPN*	IoU value for ERPN foreground anchor boxes	[0.6,1]
*IoU_ background_ERPN*	IoU value for ERPN background anchor boxes	[0,0.3)
*NMS_IoU*	Threshold value of IoU for NMS method	0.75

**Table 7:** Parameters of Faster R-CNN.

Parameter	Description	Value
*base_lr*	Initial value of the learning rate	0.001
*lr_policy*	Learning rate policy: drop the learning rate in steps by a factor of *gamma* every *stepsize* iterations	"step"
*gamma*	Factor of dropped learning rate	0.1
*stepsize*	Drop the learning rate every *stepsize* iterations	50000
*momentum*	Weight of the previous update	0.9
*weight_decay*	Factor of the regularization	0.0005
*max_iter_rpn*	Number of RPN execution steps	80000
*IoU_ foreground_RPN*	IoU value for RPN foreground anchor boxes	[0.7,1]
*IoU_ background_RPN*	IoU value for RPN background anchor boxes	[0,0.3)
*NMS_IoU*	Threshold value of IoU for NMS method	0.7
*max_iter_fastrcnn*	Number of Fast R-CNN execution steps	40000
*IoU_ foreground_fastrcnn*	IoU value for Fast R-CNN foreground anchor boxes	[0.5,1]
*IoU_ background_fastrcnn*	IoU value for Fast R-CNN background anchor boxes	[0.1,0.5)

**Table 8:** Parameters of HyperNet.

Parameter	Description	Value
*base_lr*	Initial value of the learning rate	0.001
*lr_policy*	Learning rate policy: drop the learning rate in steps by a factor of *gamma* every *stepsize* iterations	"step"
*gamma*	Factor of dropped learning rate	0.1
*stepsize*	Drop the learning rate every *stepsize* iterations	50000
*momentum*	Weight of the previous update	0.9
*weight_decay*	Factor of the regularization	0.0005
*max_iter_rpg*	Number of region proposal generation execution steps	60000
*max_iter_detection*	Number of detection execution steps	40000
*IoU_ foreground*	IoU value for foreground anchor boxes	[0.45,1]
*IoU_ background*	IoU value for background anchor boxes	[0,0.3)
*NMS_IoU*	Threshold value of IoU for NMS method	0.7

### Experiments on PASCAL VOC 2007

In this part, the comparative experiment is executed on the PASCAL VOC 2007 data set which consists of about 5k trainval images and 5k test images over 20 categories. Furthermore, the union set of VOC 2012 trainval and VOC 2007 trainval is trained by all 6 object detection methods. Next, these methods are evaluated over the VOC 2007 test set. The detailed experiment results are showed in Table 9. In order to show the advantages of the improvements in ERPN, the improvement of novel anchor boxes is applied in ERPN^a^. Furthermore, the ERPN^b^ is designed by integrating the DFPN and improved classification loss function with ERPN^a^. Finally, the PSO-SVM classifier is developed in ERPN based on ERPN^b^.

**Table 9:** Detection results on PASCAL VOC 2007 test set, the best AP of each object category and mAP are bold-faced.

Approach	mAP	aero	bike	bird	boat	bottle	bus	car	cat	chair	cow	table	dog	horse	mbike	person	plant	sheep	sofa	train	tv
Fast R-CNN	70.0	77.0	78.1	69.3	59.4	38.3	81.6	78.6	86.7	42.8	78.8	68.9	84.7	82.0	76.6	69.9	31.8	70.1	74.8	80.4	70.4
Faster R-CNN	73.2	76.5	79.0	70.9	65.5	52.1	83.1	84.7	86.4	52.0	81.9	65.7	84.8	84.6	77.5	76.7	38.8	73.6	73.9	83.0	72.6
MR-CNN	78.2	**80.3**	84.1	78.5	**70.8**	68.5	**88.0**	85.9	**87.8**	60.3	**85.2**	73.7	87.2	**86.5**	85.0	76.4	48.5	76.3	75.5	85.0	81.0
ION	75.6	79.2	83.1	77.6	65.6	54.9	85.4	85.1	87.0	54.4	80.6	73.8	85.3	82.2	82.2	74.4	47.1	75.8	72.7	84.2	80.4
HyperNet	76.3	77.4	83.3	75.0	69.1	62.4	83.1	**87.4**	87.4	57.1	79.8	71.4	85.1	85.1	80.0	**79.1**	51.2	**79.1**	75.7	80.9	76.5
ERPN^a^	74.6	78.2	81.7	72.4	67.2	53.8	84.8	85.2	86.7	54.3	83.1	67.7	86.2	85.1	78.5	77.6	39.7	74.7	75.3	84.7	74.3
ERPN^b^	78.1	78.9	83.9	78.4	69.2	68.4	87.1	85.8	87.2	59.9	83.8	73.5	86.9	85.5	85.1	78.2	51.6	76.9	75.9	85.1	81.5
ERPN	**78.6**	79.4	**84.2**	**78.9**	69.6	**68.8**	87.4	86.2	87.6	**60.5**	84.2	**74.1**	**87.4**	85.8	**85.3**	78.6	**51.9**	77.2	**76.6**	**85.4**	**82.1**

Two innovations are included in our proposed anchor boxes. Firstly the anchor boxes with 4 scales are divided into two types. The areas for one type of anchor box scales are 150^2^, 300^2^, 450^2^ and 550^2^ pixels which are suitable for the larger object detection. Therefore, ERPN^a^ achieves excellent AP on detection of bus, aero plane, horse and so on in Table 9. The areas for another type of anchor box scales are 50^2^, 100^2^, 250^2^ and 400^2^ pixels which are suitable for the smaller object detection. Thereafter, ERPN^a^ obtains good AP on detection of bird, bottle, plant and so on in Table 9. Specially, the aspect ratio of each anchor box is set the same as the aspect ratio of corresponding feature map. In other words, the improved anchor boxes are adaptively matched to the input image. From Table 9, we can see that the AP of each category for ERPN^a^ is better than that of Faster R-CNN. Simultaneously, the mAP of ERPN^a^ is higher than that of Faster R-CNN. Consequently, our proposed anchor boxes are effective. Furthermore, DFPN is used to enhance the top-level features with the context information. Particularly, a synthesized pooling method including max pooling and average pooling strategies is applied to boost the ability of pooling layers. Besides, the improved classification loss function is introduced, thus the performance of multi-task loss function is promoted. As shown in Table 9, we can see that the mAP of ERPN^b^ is higher than that of Fast R-CNN, Faster R-CNN, ION and HyperNet. Thereupon, the detection ability is enhanced by using DFPN and the improved classification loss function. The SVM classifier is used in ERPN. The parameters of SVM is optimized by PSO, therefore the classification ability of ERPN is strengthened. Additionally, ERPN contains the advantages of ERPN^a^ and ERPN^b^. Thereupon, ERPN achieves the best detection results on the categories of bike, bird, bottle, chair, table, dog, mbike, plant, sofa, train and tv. Especially, the mAP of ERPN is better than the other methods. As a result, the object detection performance of ERPN is promoted based on the improved anchor boxes and DFPN.

### Experiments on PASCAL VOC 2012

In this section, we conduct comparative experiment on the PASCAL VOC 2012 data set. Meanwhile, the experimental training data is constructed by the dataset of VOC 2007 and VOC 2012. From Table 10, we can see that Fast R-CNN achieves the highest AP for cat detection. Simultaneously, MR-CNN gets the highest AP for aero plane, boat, bus and horse detection. Additionally, HyperNet obtains the highest AP for car and sheep detection. Specially, ERPN achieves the highest AP for the rest of the object detection. Furthermore, ERPN^a^ acquires 71.9% mAP which is higher than Fast R-CNN, Faster R-CNN. Besides, ERPN^b^ gains 74.1% mAP which is higher than Fast R-CNN, Faster R-CNN, MR-CNN, ION and HyperNet. Particularly, ERPN achieves the 74.4% mAP which is higher than the rest. Consequently, ERPN method obtains the outstanding result on slightly more challenging PASCAL VOC 2012 data set. In other words, our proposed improvements of ERPN are effective.

**Table 10:** Detection results on PASCAL VOC 2012 test set, the best AP of each object category and mAP are bold-faced.

Approach	mAP	aero	bike	bird	boat	bottle	bus	car	cat	chair	cow	table	dog	horse	mbike	person	plant	sheep	sofa	train	tv
Fast R-CNN	68.4	82.3	78.4	70.8	52.3	38.7	77.8	71.6	**89.3**	44.2	73.0	55.0	87.5	80.5	80.8	72.0	35.1	68.3	65.7	80.4	64.2
Faster R-CNN	70.4	84.9	79.8	74.3	53.9	49.8	77.5	75.9	88.5	45.6	77.1	55.3	86.9	81.7	80.9	79.6	40.1	72.6	60.9	81.2	61.5
MR-CNN	73.9	**85.5**	82.9	76.6	**57.8**	62.7	**79.4**	77.2	86.6	55.0	79.1	62.2	87.0	**83.4**	84.7	78.9	45.3	73.4	65.8	80.3	74.0
ION	71.1	83.3	80.5	71.5	56.5	53	77.4	73.8	85.8	52.6	76.8	59.1	83.9	81.3	79.3	77.2	45.7	72.6	64.2	80.1	68.1
HyperNet	71.4	84.2	78.5	73.6	55.6	53.7	78.7	**79.8**	87.7	49.6	74.9	52.1	86.0	81.7	83.3	81.8	48.6	**73.5**	59.4	79.9	65.7
ERPN^a^	71.9	85.1	81.3	75.8	54.8	51.6	78.1	76.8	88.6	47.1	78.5	58.4	87.1	82.3	82.8	79.3	45.5	72.8	64.4	81.3	65.5
ERPN^b^	74.1	84.9	82.9	76.7	57	63.2	78.5	77	88.3	54.9	78.9	62.2	87.2	82.8	84.5	79.5	48.6	73	65.7	80.9	74.2
ERPN	**74.4**	85.2	**83.3**	**77.1**	57.2	**63.4**	78.8	77.5	88.7	**55.5**	**79.4**	**62.6**	**87.4**	83	**85.2**	**79.8**	**48.9**	73.2	**66.4**	**81.6**	**74.3**

### Small objects detection

**Fig 9:**
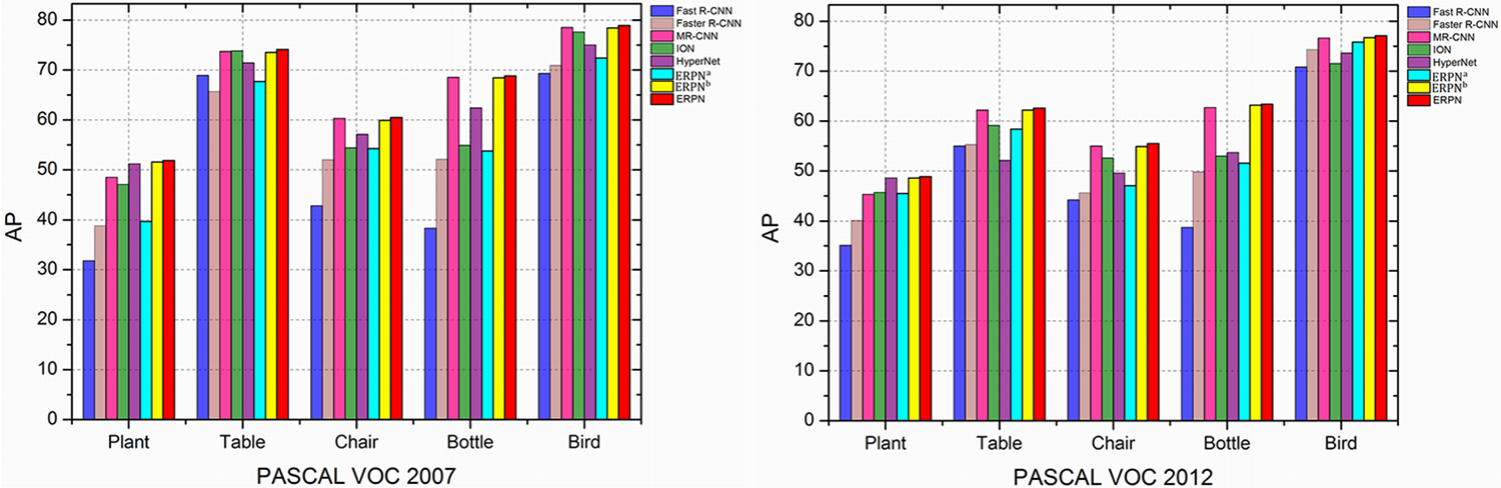
Small objects detection results on PASCAL VOC 2007 and VCO 2012 test sets.

For object detection methods, small objects detection is a challenging task. Specially, a small object will be just few pixels when it goes to the last convolutional layer of VGG16 network. The areas for one type of improved anchor box scales are 50^2^, 100^2^, 250^2^, and 400^2^ pixels. Because the areas of 50^2^ and 100^2^ are small, therefore the smaller objects are easy to be found. Furthermore, our proposed DFPN is applied to enhance the top-level features with the context information. In order to integrate lower feature maps with the higher feature maps, deconvolutional layer [40] (Deconv) is introduced into DFPN. Deconv is applied to increase the resolution of the higher convolutional feature maps. Particularly, a synthesized pooling method including max pooling and average pooling strategies is applied to boost the ability of pooling layers. For a resized 1000 × 600 input image, the feature map resolution of DFPN is 125 × 75. Therefore, the increased resolution of final output feature maps is suitable for small object detection. Potted plant, table, chair, bottle and bird are typical small objects in VOC 2007 and VOC 2012 test sets. From Fig 9, we can see that AP of ERPN^b^ on detecting potted plant, table, chair, bottle and bird is higher than Fast R-CNN, Faster R-CNN, ION, MR-CNN and HyperNet. Moreover, the AP of ERPN on detecting these small objects is the best. As a result, the ability of ERPN for small object detection is enhanced by applying our improvements.

### Analysis of Recall-to-IoU

The recall of state-of-the-art object detection methods with *N* proposals at different IoU ratios is calculated on PASCAL VOC 2007 test set. The number of proposals is the top-N ranked ones based on the confidence generated by the object detection methods. Simultaneously, *N* is assigned to 200, 500 and 1000 respectively. IoU is defined as (*w*∩*b*)/(*w*∪*b*) where *w* and *b* are the object proposal bounding boxes and ground truth boxes. Because the adjacent proposed anchor boxes have different areas which are [150^2^, 300^2^, 450^2^, 550^2^] and [50^2^, 100^2^, 250^2^, 400^2^], thus the IoU for adjacent proposed anchor boxes is lower than the IoU for original anchor boxes. As a result, the number of available proposals is more than that of original anchor boxes when the value of IoU is high.

**Fig 10:**
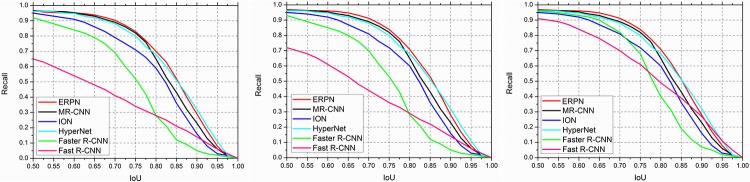
Recall versus IoU threshold on the PASCAL VOC 2007 test set. Left: 200 region proposals. Middle: 500 region proposals. Right: 1000 region proposals.

From Fig 10, we can see that the ERPN method works very well when the number of proposals drops from 1000 to 200. Therefore, the number of proposals *N* is set to 200 for ERPN. Moreover, the recall of ERPN is better than other methods across a variety of IoU thresholds, especially when the IoU threshold is high (e.g., > 0.7). Consequently, the IoU threshold for NMS is fixed at 0.75 in ERPN. As a result, the experiment shows that the ability of object detection is enhanced by our proposed ERPN.

### Experiments on MS COCO

In this part, the experiment is executed on the MS COCO data set which consists of about 80k training images and 40k validation images over 80 categories. The experiment results are implemented on the standard test set (test-std). Comparing to the PASCAL VOC metric which only requires IoU of 0.5, the mAP is averaged over different IoU thresholds on the MS COCO data set.

The results are presented in Table 11. The mAP@[0.5:0.95] of our ERPN^a^ is 24.1% which is better than Fast R-CNN (19.3%) and Faster R-CNN (21.9%). In other words, the range of object detection is strengthened by applying the interspersed anchor boxes with scales of [150^2^, 300^2^, 450^2^, 550^2^] and [50^2^, 100^2^, 250^2^, 400^2^]. Additionally, it is worth noting that our method is more accurate for small objects. Furthermore, the IoU for adjacent proposed anchor boxes is lower than the IoU for original anchor boxes. Thereafter, the number of available proposals is more than that of original anchor boxes. Specially, the aspect ratio of each anchor box is adapted to the shape of image. Consequently, the performance of object detection of ERPN^a^ is promoted. Moreover, our proposed DFPN is applied to enhance the top-level features with the context information. Particularly, a synthesized pooling method including max pooling and average pooling strategies is applied to boost the ability of pooling layers. Besides, the improved classification loss function is used in ERPN^b^, thereafter the convergence capability of ERPN^b^ is enhanced. The mAP@[0.5:0.95] of ERPN^b^ is 30.9% which is better than Fast R-CNN (19.3%), Faster R-CNN (21.9%) and ION(30.7%). Finally, the improved classification loss function is used in ERPN, thereafter the convergence capability of ERPN is enhanced. Finally, because the PSO-SVM classifier is applied to ERPN, thereupon the classification ability of ERPN is boosted. The mAP@[0.5:0.95] of ERPN is 31.7% which is the best. As a result, our proposed ERPN achieves outstanding detection results on MS COCO data set.

**Table 11:** Detection results on MS COCO test-std. The best result is bold-faced. A: improved anchor boxes, D: DFPN.

Method	A	D	Avg.Precision,IoU:			Avg.Precision,Area:			Avg.Recall,#Dets:			Avg.Recall,Area:		
			0.5:0.95	0.50	0.75	Small	Med.	Large	1	10	100	Small	Med.	Large
Fast R-CNN			19.3	39.3	19.9	3.5	18.8	34.6	21.4	29.5	29.8	7.7	32.2	50.2
Faster R-CNN			21.9	42.7	22.3	4.2	19.3	36.1	22.5	31.2	31.4	8.9	34.2	52.3
ION			30.7	52.9	31.7	11.8	32.8	44.8	27.7	42.8	45.4	23.0	50.1	63.0
ERPN^a^	√		24.1	45.1	24.5	6.6	21.4	38.4	24.8	33.5	33.8	11.3	36.4	54.7
ERPN^b^		√	30.9	52.9	32.2	12.9	32.8	44.9	28.1	43.5	45.9	23.5	50.6	63.3
ERPN	√	√	**31.7**	**53.5**	**32.9**	**13.5**	**33.4**	**45.6**	**28.5**	**43.8**	**46.4**	**24.5**	**51.5**	**64.3**

### Comparison of classifiers

Softmax classifier is used in RPN of Faster R-CNN to solve the classification problem. However, SVM classifier is taken by our novle ERPN. In order to compare the SVM, softmax and PSO-SVM classifiers, following experiments are carried out to evaluate the classifiers on PASCAL VOC 2012 test set.

From Table 12 we can see that the mAP of ERPN with softmax is 78.1% which is 0.4 point higher than the mAP of ERPN with SVM. The parameters of RBF kernel function for SVM are selected by grid search method. Nevertheless, the global searching ability of grid search is not good. Thereafter, the classification rate of SVM is easily to fall into local optima by using grid search method. In other words, the performance of SVM is seriously affected by the parameters *C* and *γ*. In order to enhance the ability of SVM, the PSO method is applied to optimize the parameters *C* and *γ* of SVM in ERPN. The particles of PSO represent the changes in parameters C and *γ*. From Table 12 we can find that the mAP of ERPN with PSO-SVM is 78.6% which is the best. As a result, the classification ability of ERPN is strengthened based on PSO-SVM.

**Table 12:** Comparison between SVM and softmax classifiers.

Classifier	mAP	aero	bike	bird	boat	bottle	bus	car	cat	chair	cow	talbe	dog	horse	mbike	person	plant	sheep	sofa	train	tv
SVM	77.7	78.3	83.4	78.2	68.9	68.1	86.5	85.2	86.9	59.7	83.1	73.3	86.2	85.3	84.5	77.7	51.4	76.7	75.3	84.5	81.4
Softmax	78.1	78.9	83.9	78.4	69.2	68.4	87.1	85.8	87.2	59.9	83.8	73.5	86.9	85.5	85.1	78.2	51.6	76.9	75.9	85.1	81.5
PSO-SVM	**78.6**	**79.4**	**84.2**	**78.9**	**69.6**	**68.8**	**87.4**	**86.2**	**87.6**	**60.5**	**84.2**	**74.1**	**87.4**	**85.8**	**85.3**	**78.6**	**51.9**	**77.2**	**76.6**	**85.4**	**82.1**

### Analysis of improved classification loss function

In this part, the loss functions of RPN and ERPN are compared on VOC 2007, VOC 2012 and MS COCO data sets. The *L*_*cls*_(RPN) represents the loss function of ERPN is replaced by Eq (15). Our improved classification loss function is used in the *L*_*cls*_(ERPN). From Table 13 we can find that the *L*_*cls*_(RPN) achieves 78.2%, 73.9% and 31.3% mAPs on on VOC 2007, VOC 2012 and MS COCO data sets. However, the performance of *L*_*cls*_(ERPN) is better than *L*_*cls*_(RPN). Because novel coefficients of improved classification loss function are developed, thereafter the training of negative samples is strengthened. In other words, the balance of the training samples has been improved. As a result, the performance of multi-task loss function is promoted.

**Table 13:** Experiment results for improved loss function.

Data set	VOC 2007	VOC 2012	MS COCO
*L*_*cls*_(RPN)	78.2	73.9	31.3
*L*_*cls*_(ERPN)	78.6	74.4	31.7

**Fig 11:**
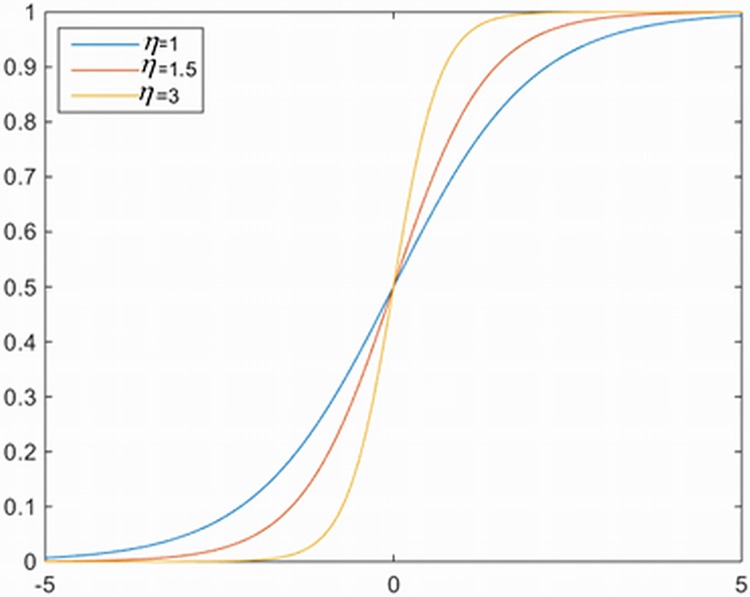
The influence for Eta on coefficients of improved classification loss function.

Because the novel coefficients of improved classification loss function are sigmoid function, therefore the curve of sigmoid (*η*^*x*^) is influenced by value of *η*. If the value of *η* is too large, then the curve of sigmoid function changes very seriously. Therefore the effect of positive samples is reduced too much. If the value of *η* is too small, the effect of positive samples is enlarged, thereafter the problem of imbalance training is not solved. The appropriate value of *η* is important. Different value of *η* is selected in Fig 11. The curve of sigmoid function satisfies our requirements when *η* is assigned to 1.5. Because the curve of sigmoid function is not changed very violently or very slowly when *η* is assigned to 1.5, therefore this curve satisfies our requirements. Moreover, the ERPN achieves the best mAP on VOC 2007, VOC 2012 and MS COCO data sets when *η* = 1.5. As a result, the convergence effect of loss function is enhanced by using the variable *η*.

### Running time

Because 4 anchor boxes are applied to each sliding position. Therefore, total around 30k anchor boxes are generated for a convolutional feature map of a size *W*×*H* (around 9k). However, output layer of DFPN has 1.2 × 10^4^ parameters (512 × (4 + 2) × 4 for VGG-16) which are less than that of RPN. Thereupon, the computation speed of object detection is accelerated. Moreover, 200 top ranked proposals are generated after NMS. As a result, the computation speed of object detection is accelerated. From Table 14, we can see that our ERPN-based Faster R-CNN has a frame rate of 5.8 fps on a single NVIDIA TitanX GPU by applying VGG-16 network. Meanwhile, the detection speed of ERPN is faster than other methods.

**Table 14:** Detection speed of different methods on the PASCAL VOC 2007 test set.

Approach	Fast R-CNN	Faster R-CNN	ION	MR-CNN	HyperNet	ERPN
Frame rate (fps)	0.5	5	1.25	2	5	5.8

## Conclusion

In this paper, a state-of-the-art region proposal generation architecture ERPN is proposed. Context information is integrated with the output convolutional features based on DFPN. Moreover, novel anchor boxes are designed with interspersed scales and adaptive aspect ratios. Additionally, PSO-SVM is developed as the classifier of ERPN. Finally, the classification part of multi-task loss function in RPN is improved. Our proposed ERPN obtains excellent object detection on PASCAL VOC 2007, 2012 and MS COCO data sets, surpassing other five object detection methods in terms of both speed and accuracy.

## Supporting information

S1 Table PASCAL VOC 2007, PASCAL VOC 2012 and MS COCO data set information. (DOCX)
