# Supplementary material for: An Enhanced Region Proposal Network for object detection using deep learning method
Source: PLoS One. 2018 Sep 20;13(9):e0203897. doi: 10.1371/journal.pone.0203897 (PMC6147513; doi:10.1371/journal.pone.0203897)
Supplement: S1 Table — (DOCX) [file pone.0203897.s001.docx]

**Supporting Information**

PASCAL VOC 2007

http://host.robots.ox.ac.uk/pascal/VOC/voc2007/index.html

The goal of this challenge is to recognize objects from a number of visual object classes in realistic scenes (i.e. not pre-segmented objects). It is fundamentally a supervised learning problem in that a training set of labeled images is provided. The twenty object classes that have been selected are:

- *Person:* person
- *Animal:* bird, cat, cow, dog, horse, sheep
- *Vehicle:* aeroplane, bicycle, boat, bus, car, motorbike, train
- *Indoor:* bottle, chair, dining table, potted plant, sofa, tv/monitor

The training data provided consists of a set of images; each image has an annotation file giving a bounding box and object class label for each object in one of the twenty classes present in the image. Note that multiple objects from multiple classes may be present in the same image. Some [example images](http://host.robots.ox.ac.uk/pascal/VOC/voc2007/examples/index.html) can be viewed online.

Annotation was performed according to a set of guidelines distributed to all annotators. The data will be made available in two stages; in the first stage, a development kit will be released consisting of training and validation data, plus evaluation software (written in MATLAB). One purpose of the validation set is to demonstrate how the evaluation software works ahead of the competition submission.

In the second stage, the test set will be made available for the actual competition. As in the VOC2006 challenge, no ground truth for the test data will be released until after the challenge is complete.

The data has been split into 50% for training/validation and 50% for testing. The distributions of images and objects by class are approximately equal across the training/validation and test sets. In total there are 9,963 images, containing 24,640 annotated objects.

**Table 1. PASCAL VOC 2007 information**

| No. | Data set | No. of classes | No. of images | No. of annotated objects |
| --- | --- | --- | --- | --- |
| 1 | PASCAL VOC 2007 | 20 | 9963 | 24640 |

PASCAL VOC 2012

<http://host.robots.ox.ac.uk/pascal/VOC/voc2012/index.html>

The data will be made available in two stages; in the first stage, a development kit will be released consisting of training and validation data, plus evaluation software (written in MATLAB). One purpose of the validation set is to demonstrate how the evaluation software works ahead of the competition submission.

In the second stage, the test set will be made available for the actual competition. As in the VOC2008-2011 challenges, no ground truth for the test data will be released.

The data has been split into 50% for training/validation and 50% for testing. The distributions of images and objects by class are approximately equal across the training/validation and test sets. [Statistics](http://host.robots.ox.ac.uk/pascal/VOC/voc2012/dbstats.html) of the database are online.

**Table 2. PASCAL VOC 2012 information**

| No. | Data set | No. of classes | No. of images | No. of annotated objects |
| --- | --- | --- | --- | --- |
| 1 | PASCAL VOC 2012 | 20 | 11530 | 27450 |

MS COCO

<http://cocodataset.org/>

The COCO Object Detection Task is designed to push the state of the art in object detection forward. COCO features two object detection tasks: using either bounding box output or object segmentation output. The COCO train, validation, and test sets, containing more than 200,000 images and 80 object categories, are available on the [download](http://cocodataset.org/#download) page. All object instances are annotated with a detailed segmentation mask. Annotations on the training and validation sets (with over 500,000 object instances segmented) are publicly available.

**Table 3. MS COCO information**

| No. | Data set | No. of classes | No. of images | No. of annotated objects |
| --- | --- | --- | --- | --- |
| 1 | MS COCO 2015 | 80 | 328000 | 2500000 |
